# Crosstalk of RNA methylation writers defines tumor microenvironment and alisertib resistance in breast cancer

**DOI:** 10.3389/fendo.2023.1166939

**Published:** 2023-09-25

**Authors:** Xiaoqiang Zhang, Li Shen, Yanhui Zhu, Changyuan Zhai, Hanling Zeng, Xiaoan Liu, Jing Tao

**Affiliations:** ^1^ Department of General Surgery, The Fourth Affiliated Hospital of Nanjing Medical University, Nanjing Medical University, Nanjing, Jiangsu, China; ^2^ Breast Disease Center, The First Affiliated Hospital of Nanjing Medical University, Nanjing, Jiangsu, China

**Keywords:** breast cancer, RNA methylation modification, RMW_Score, prognosis, TCP1, alisertib

## Abstract

**Background:**

The five major RNA methylation modifications (m6A, m1A, m6Am, m5C, and m7G) exert biological roles in tumorigenicity and immune response, mediated mainly by “writer” enzymes. Here, the prognostic values of the “writer” enzymes and the TCP1 role in drug resistance in breast cancer (BC) were explored for further therapeutic strategies.

**Methods:**

We comprehensively characterized clinical, molecular, and genetic features of subtypes by consensus clustering. RNA methylation modification “Writers” and related genes_risk (RMW_risk) model for BC was constructed *via* a machine learning approach. Moreover, we performed a systematical analysis for characteristics of the tumor microenvironment (TME), alisertib sensitivity, and immunotherapy response. A series of experiments *in vitro* were carried out to assess the association of TCP1 with drug resistance.

**Results:**

One “writer” (RBM15B) and two related genes (TCP1 and ANKRD36) were identified for prognostic model construction, validated by GSE1456, GSE7390, and GSE20685 cohorts and our follow-up data. Based on the patterns of the genes related to prognosis, patients were classified into RMW_risk-high and RMW_risk-low subtypes. Lower RMW_Score was associated with better overall survival and the infiltration of immune cells such as memory B cells. Further analysis revealed that RMW_Score presented potential values in predicting drug sensitivity and response for chemo- and immunotherapy. In addition, TCP1 was confirmed to promote BC alisertib-resistant cell proliferation and migration *in vitro*.

**Conclusion:**

RMW_Score could function as a robust biomarker for predicting BC patient survival and therapeutic benefits. This research revealed a potential TCP1 role regarding alisertib resistance in BC, providing new sights into more effective therapeutic plans.

## 1 Introduction

Breast cancer (BC) is a complex disease affected by multiple risk factors, including genetic and environmental factors, recognized as the most common cause of cancer-related deaths among women worldwide (1–3). Recent studies have focused on investigating the underlying association of the tumor microenvironment (TME) with BC. The TME, composed of tumor cells and multiple non-malignant cell types (immune cells, fibroblasts, and endothelial cells), exerts influences at all disease stages, including tumor initiation, metastatic progression, and response to therapies (4–6). Evidence indicates that significant differences could be derived from various cellular compositions of immune infiltration in BC, thus offering prognostic and predictive values (7). In D. Hammerl and T. Karn’s investigations, tumor-infiltrating leukocytes (TILs) could serve as a sensitive biomarker, a high level of which indicated better survival, especially in patients with triple-negative breast cancer (TNBC) (8, 9). However, apart from the apparent progress achieved in immunotherapies, clinical benefits from these applications are restricted to a minority of patients (10). TILs and other components of the TME in BC were demonstrated to predict response to anticancer immunotherapies (11). Thus, a comprehensive understanding of the TME in BC will assist in enhancing clinical prediction and developing effective individual strategies.

Recent years have witnessed the identification of over 100 types of RNA modifications including methylations, cytosine modifications, isomerization of uridine, and ribose modification (12, 13). Among them, RNA methylation modifications are involved in the pathogenesis of various diseases including cancer through the dysregulation of epigenetic pathways (14, 15). Here, most attention has been focused on five major RNA methylation modifications, i.e., *N* ^6^-methyladenosine (m^6^A), *N* ^1^-methyladenosine (m^1^A), *N* ^6^,2′-*O*-dimethyladenosine (m^6^Am), 5-methylcytosine (m^5^C), and 7-methylguanosine (m^7^G), reckoned to be activated by “writers” enzymes (16–23). The valid associations of the five major RNA modifications and related enzymes with the TME are reported in recent studies (24–26). However, given only one or two RNA modification “writers” contained in these studies, the antitumor effect of RNA modifications through multiple regulators lacks attention, especially in BC. Thus, our study sheds light on the correlations between numerous “writers” and the TME in BC for a holistic understanding.

Alisertib (MLN8237), an Aurora A inhibitor, has been shown to prevent Aurora A from being phosphorylated. This drug has been reported to prevent N-myc signaling and tumor growth by inhibiting the interaction between N-myc and the factor AURKA (27). By modifying the expression of the BCL-2 family (Bcl-xL), the drug might cause apoptosis (28). In a randomized clinical trial, the safety and effectiveness of weekly paclitaxel therapy with alisertib addition in metastatic breast cancer patients were assessed (29). This phase 2 study found that compared with paclitaxel, weekly paclitaxel plus oral alisertib presented improved progression-free survival (PFS) significantly, and the toxic impacts were manageable. Nevertheless, studies concerning the potential mechanism of BC cells resistant to alisertib remain few.

In this work, the prognostic roles of RNA methylation modification “writers” were explored using bioinformatics and statistical analysis, based on cases from The Cancer Genome Atlas (TCGA), the Gene Expression Omnibus (GEO) dataset, and our follow-up data. One “writer” and two related genes were identified to construct the RNA methylation modification “writers” and related genes_risk score (RMW_Score) model. It was observed that the risk signature was related to immune infiltration, therapeutic response, and genetic and molecular features. Interestingly, we also proposed the TCP1 role in resistance to alisertib, which may offer a novel target for overcoming drug resistance.

## 2 Materials and methods

### 2.1 Data collection and processing

Complete clinical information and gene expression data of BC patients were extracted from TCGA and GEO databases. With the use of the “caret” R package, the 1,089 BC patients from TCGA database (https://portal.gdc.cancer.gov/) were randomly assigned to training and validation cohorts in a 1:1 ratio. GSE1456, GSE7390, and GSE20685 cohorts were retrieved from the GEO database (https://www.ncbi.nlm.nih.gov/geo/). A total of 194 BC samples from our follow-up data were used for further validation. The IMvigor210 cohort was included to evaluate the role of RMW_Score in predicting immunotherapy benefits. The expression of data and clinical details of the IMvigor210 cohort were obtained from http://research-pub.gene.com/IMvigor210CoreBiologies.

### 2.2 Clustering pattern

Unsupervised clustering algorithm was performed to identify the robust clustering of BC using the “ConsensusClusterPlus” package. According to previously published literature, 20 acknowledged RNA methylation modification “writers” were collated to explore potential prognostic biomarkers. These “writers” consist of seven m^6^A enzymes (METTL14, METTL16, WTAP, RBM15, RBM15B, ZC3H13, and VIRMA), five m^1^A enzymes (TRMT61A, TRMT61B, TRMT10C, TRMT6, and TRPM5), one m^6^Am enzyme (PCIF1), five m^5^C enzymes (NSUN2, NSUN3, NSUN4, NSUN5, and NSUN6), and two m^7^G enzymes (METTL1 and WDR4).

### 2.3 Enrichment analysis

We identified differentially expressed genes (DEGs) *via* Bioconductor packages “DESeq2” and “limma”. “GSEA” and “GSVA” R packages were utilized respectively for gene set enrichment analysis (GSEA) and gene set variation analysis (GSVA) to study the variation of RNA modification patterns in biological processes. The gene sets were available from the MSigDB database.

### 2.4 Immune infiltration

The infiltrating level of 28 immune cell types was quantified using single-sample gene set enrichment analysis (ssGSEA). ssGSEA employed an enrichment score to represent the relative abundance of each immune cell type by package “gsva”. The normalized distribution was from 0 to 1. *Via* TIMER, CIBERSORT, and xCell algorithms, the levels of immune infiltrating cells were calculated. The related data were downloaded from the TIMER2.0 website (http://timer.comp-genomics.org/).

### 2.5 Construction of the RMW_Score and drug sensitivity

The RMW_Score of each BC patient was calculated by the formula Risk score = $\sum_{i=1}^{n}\left(Coef_{i}*X_{i}\right)$ , where *i* represents the RNA modification phenotype-related genes and *X_i_* represents the expression value of each gene. *Coef_i_* is the coefficient of each gene in the RMW_Score model. The *Coef_i_* for RBM15B, TCP1, and ANKRD36 were −0.101981953, 0.017033828, and −1.224417758, respectively. Genomics of Drug Sensitivity in Cancer (GDSC; http://www.cancerrxgene.org/downloads) provided the antitumor drugs in cancer cell lines and relative drug targets/pathways for investigation. Spearman’s correlation analysis was applied to calculate the correlation of RMW_Score with drug sensitivity (|Rs| > 0.2 and false discovery rate (FDR)<0.05).

### 2.6 Cell culture and cell transfection

The MDA-MB-231, BT-549, alisertib-resistant MDA-MB-231, and BT-549 cells were cultured in Dulbecco’s modified Eagle’s medium (DMEM) (Gibco, Grand Island, NY, USA) medium containing 10% fetal bovine serum (FBS; Gibco) and 1% penicillin/streptomycin (Gibco, USA) at 37°C in a 5% CO_2_ incubator. MDA-MB-231/alisertib and BT-549/alisertib cells were also cultured in 2 μg/ml of alisertib to maintain drug resistance. Small interfering RNA (siRNA) (GenePharma, Shanghai, China) was utilized to silence transiently the expression of TCP1. Based on the producer’s instructions, we conducted transfection with Lipofectamine 2000 (Invitrogen, Carlsbad, CA, USA).

### 2.7 Alisertib-sensitive assay

Cell Counting Kit-8 (CCK-8) was used to determine the half-maximal inhibitory concentration (IC50) for the drug-sensitivity test to alisertib. Fresh media were used to cultivate 1.5 * 10^4^ MDA-MB-231 and BT-549 cells in 96-well plates. The corresponding concentration of alisertib (0.1, 0.5, 1, 5, 10, 20, and 40 μM) was administrated to cells. Cell Counting Kit-8 (Dojindo, Japan) was used at the specified time to measure the *in vitro* drug sensitivity using a fluorometric microplate reader (Thermo Fisher Scientific, Waltham, MA, USA) set at 450 nm. With GraphPad 8.0, the IC50 was visually determined.

### 2.8 Quantitative reverse transcription polymerase reaction

TRIzol reagent (Invitrogen, USA) was used to extract total RNA from cells in accordance with the producer’s protocol. RNA was reverse transcribed into cDNA through a reverse transcription kit (Takara, Maebashi, Japan). The primers sequences were listed as follows: GAPDH-F: 5′-GACAGTCAGCCGCATCTTCT-3′; GAPDH-R: 5′-TTAAAAGCAGCCCTGGTGAC-3′; TCP1-F: 5′-CGGGATCCATGGCGGTGAAGGCCCTT-3′; TCP1-R: 5′-GCTTCTAGATCAGCCTTTAAGAGATGAC-3′.

### 2.9 EdU assay

BeyoClick™ EdU Cell Proliferation Kit with Alexa Fluor 594 (Beyotime, Shanghai, China) was utilized. Cells were washed with phosphate-buffered saline (PBS). DMEM (Gibco, USA) and 10 μM of EdU were then added to the plate. After 2-h incubation at 37°C/5% CO_2_, the medium containing DMEM and EdU was removed by washing the cells with PBS. A solution of 4% paraformaldehyde was used as a fixative solution at room temperature for 30 min. The cells were then stained with DAPI for 3 min. At last, the cells were washed with PBS and observed with an inverted microscope.

### 2.10 Cell proliferation assay

MDA-MB-231/alisertib cells receiving si-NC or si-TCP1 treatment were seeded in 96-well plates. CCK-8 solution (RiboBio, Guangzhou, China) measuring 10 μl was added per well at a specified time (0, 24, 48 h, etc.) based on the protocol. We used a microplate reading element (Synergy4, USA) to measure the cell absorbance at 450 nm.

### 2.11 Transwell assay

The methods of the migration assay were similar to those of the invasion assay except for the membrane type in the upper transwell. The membrane for migration detection was normal, while that for invasion detection was a Matrigel-coated membrane (BD Biosciences, San Jose, CA, USA). MDA-MB-231/alisertib cells receiving si-NC or si-TCP1 were cultured with serum-free DMEM medium in the upper chamber. A complete medium including 10% FBS was placed into the bottom chamber as a chemotactic agent. After 48-h incubation, the cells were fixed with 4% paraformaldehyde and then stained with crystal violet. At last, we used a microscope to observe the difference.

### 2.12 Colony formation assay

The single-layer culture cell suspension during the logarithmic growth phase was diluted due to multiple gradients, and the culture plate was inoculated with the appropriate cell density. After the supernatant was discarded, PBS was used to wash the cells twice. Pure methanol measuring 5 mL was added at room temperature for 15 min to fix cells. After removal of the fixative solution, Giemsa stain was added to establish a staining solution for 10–30 min. Finally, the plate was turned upside down with a grid of transparencies overlaying, and the clones were directly counted by the naked eye.

### 2.13 Western blotting

radioimmunoprecipitation assay (RIPA) buffer (Beyotime, China) with protease inhibitors (Sigma-Aldrich, St. Louis, MO, USA) was used to lyse the cells. The protein lysates were separated by the 10% sodium dodecyl sulfate–polyacrylamide gel electrophoresis (SDS-PAGE) and then transferred by polyvinylidene fluoride (PVDF) membranes (Millipore, Billerica, MA, USA). Membranes were exposed under primary (anti-TCP1 antibody, Proteintech, Wuhan, China; anti-α-tubulin antibody, Proteintech, China) and secondary antibody incubation. After washing, the chemiluminescence system (Bio-Rad, Hercules, CA, USA) and Image Lab Software (NIH) were respectively utilized for obtaining signals and processing them.

### 2.14 Cell cycle arrest and apoptosis measurement by flow cytometry

MDA-MB-231/alisertib cells treated differently were washed with PBS. The cells were then fixed with 75% ethanol and kept at −20°C overnight. The cell cycle detection kit (MultiSciences, Hangzhou, China) and Annexin V-APC/PI Apoptosis Detection Kit (MultiSciences, China) were used respectively for cell cycle distribution and apoptotic cell staining. The different cells were ultimately analyzed by flow cytometry.

### 2.15 Statistical analysis

Spearman’s correlation analysis was used to estimate the correlation coefficient of RNA modification “writers”. Moreover, univariate Cox regression assisted in identifying statistically significant genes related to prognostic value. The association between the RMW_Score signature and prognosis was analyzed *via* the Kaplan–Meier survival analysis with “survival” and “survminer” packages. The cutoff point of survival information was used for dichotomy RMW_Score. Then, potential points were tested to obtain the maximum rank statistic. In addition, the classification performance of the RMW_Score signature in prognosis was evaluated through the receiver operating characteristic (ROC) curve and the area under the curve (AUC) values *via* the “ROC” R package. Univariate and multivariate Cox regression were employed to demonstrate the role of RMW_Score in predicting prognosis independently. A nomogram model was constructed by the “rms” R package. All statistical analyses were performed *via* R software (Version 4.2.1), GraphPad Prism 8.0 (GraphPad Software, San Diego, CA, USA), and SPSS 26.0 (IBM, Armonk, NY, USA). Statistical significance was defined as p< 0.05.

## 3 Results

### 3.1 Consensus clustering for BC patients in TCGA cohort

**Figure 1:**
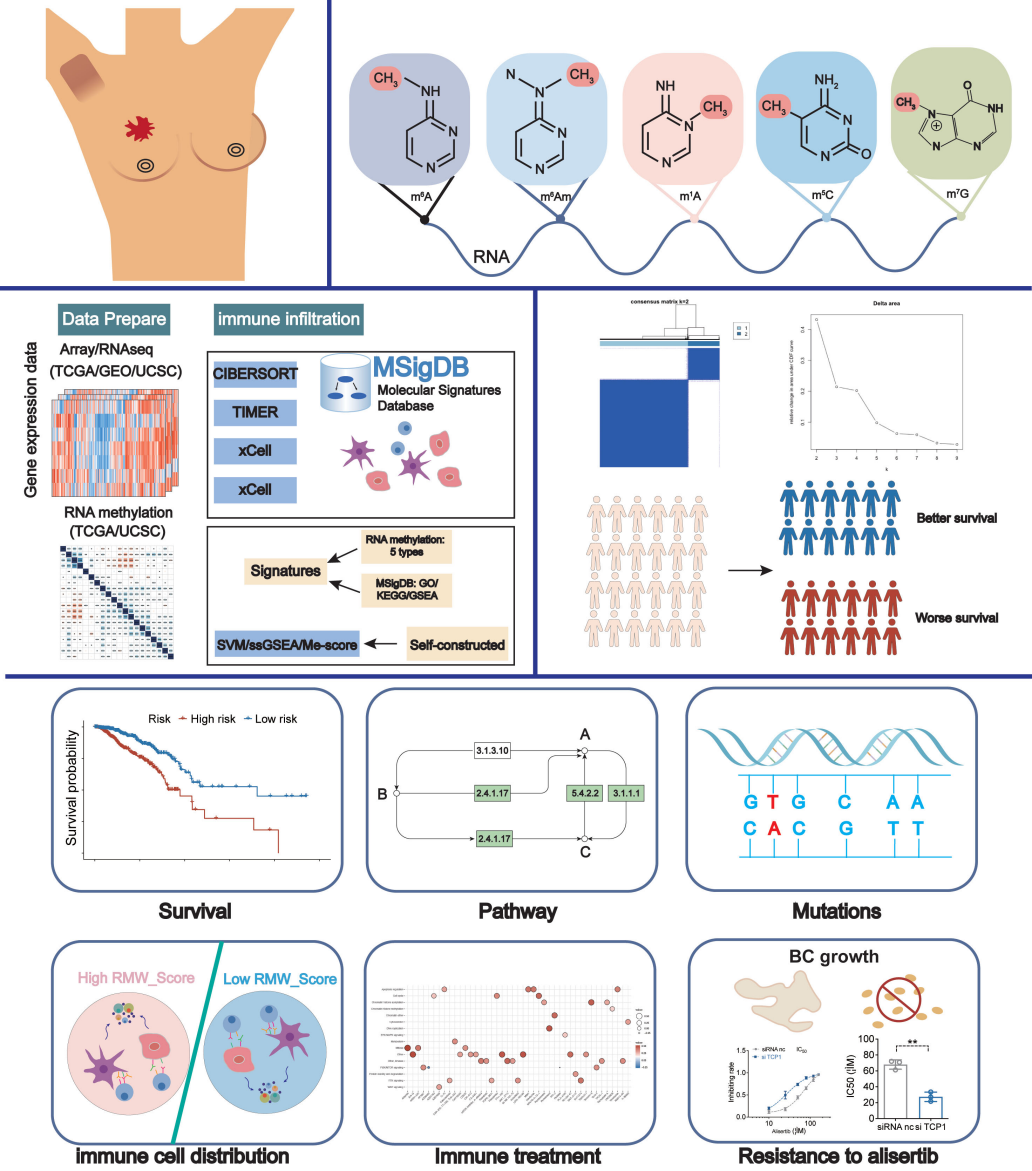
Flowchart of the study design.

**Figure 2:**
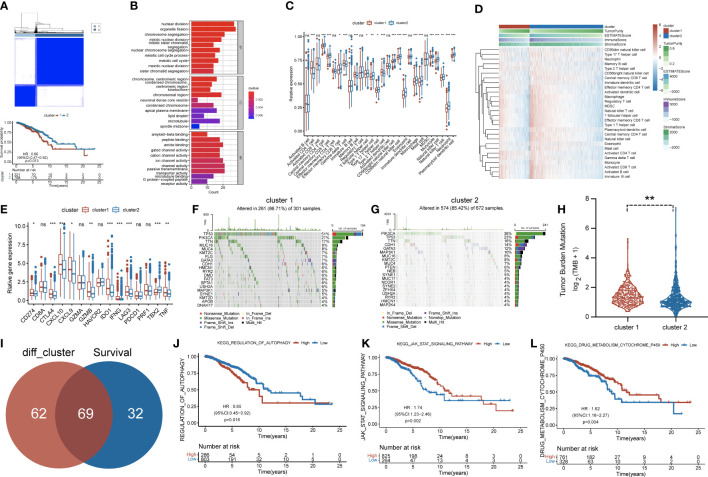
**(A)** Consensus clustering matrix for k = 2 and Kaplan–Meier curve revealed OS of patients in cluster 1 (red) and cluster 2 (blue). **(B)** Gene Ontology (GO) based on differentially expressed genes (DEGs) between two clusters. **(C)** The infiltrating levels of 28 immune cell types were compared between cluster 1 (red) and cluster 2 (blue). ns, no significance,*p < 0.05, **p < 0.01 and ***p < 0.001. **(D)** Heatmap for the landscape of immune cell types (rows) in patients (columns). Column annotations represent cluster, Tumor Purity, ESTIMATE Score, Immune Score, and Stromal Score. **(E)** The box plot compared the expression of different immune targets between cluster 1 (red) and cluster 2 (blue). ns,no significance,*p < 0.05, **p < 0.01 and ***p < 0.001. Waterfall maps revealed genetic alterations of BC patients in cluster 1 **(F)** and cluster 2 **(G)**. **(H)** Relative distribution of tumor mutational burden (TMB) in cluster 1 versus cluster 2 exhibited in the boxplot. **(I)** Venn diagram indicating 69 survival-related pathways. **(J–L)** Kaplan–Meier curves for three pathways highly associated with survival, including regulation of autophagy, JAK/STAT signaling pathway, and drug metabolism cytochrome p450. OS, overall survival; BC, breast cancer.

The flowchart of our work is shown in **Figure 1**. With consensus clustering performed based on RNA methylation modification (k = 2), 1,089 BC patients from TCGA dataset were stratified into two subgroups: cluster 1 (n = 321) and cluster 2 (n = 768, **Figure 2A**). Noticeably, the Kaplan–Meier curves revealed that patients in cluster 2 had better overall survival (OS; p = 0.013). To explain the significant difference in OS, Gene Ontology (GO) enrichment analysis was performed, showing that DEGs between the clusters were identified enriched in organelle fission, nuclear division, and amide binding (**Figure 2B**). We also observed the proportions of 28 immune cell types between clusters 1 and 2 by leveraging the ssGSEA algorithm. The distribution of 28 immune cell types is illustrated in **Figure 2C**, supporting that cluster 1 was associated with higher proportions of activated CD4 T cell, gamma delta T cell, memory B cell, and type 2 T helper cell. However, the proportions of most immune cell types were higher in cluster 2 than those in cluster 1, such as central memory CD4 T cell, central memory CD8 T cell, effector memory CD8 T cell, T follicular helper cell, type 1 T helper cell, type 17 T helper cell, CD56bright natural killer cell, CD56dim natural killer cell, eosinophil, macrophage, mast cell, monocyte, natural killer cell, natural killer T cell, neutrophil, and plasmacytoid dendritic cell. In addition, **Figure 2D** displays the distribution of immune cells in different subtypes, including clusters, tumor purity, estimate score, immune score, and stromal score.

**Figure 3:**
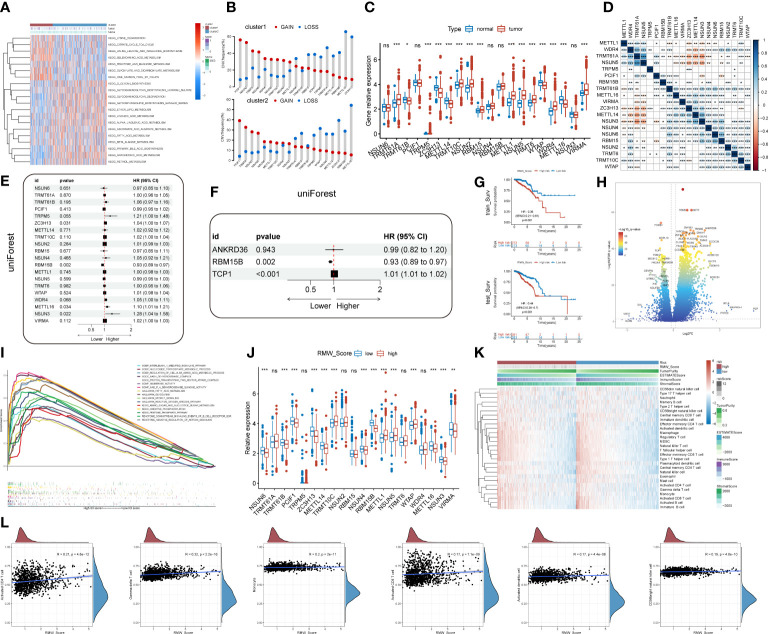
**(A)** A heatmap showing KEGG pathways (rows) for each sample (columns) in both clusters *via* gene set variation analysis (GSVA). **(B)** The CNV gain and loss frequency of RNA modification “writers” in cluster 1 and cluster 2. **(C)** Box plots compared the expression distribution of 20 “writers” between paired tumor (red) and normal (blue) tissues. ns, no significance, *p < 0.05, **p < 0.01, ***p < 0.001. **(D)** Heatmap displaying the correlations among 20 “writers” of five types of RNA methylation modification in BC by Spearman’s correlation analysis. Red, negative correlation; blue, positive correlation. ^*^p< 0.05, ^**^p< 0.01, ^***^p< 0.001. **(E, F)** Identification of prognostic “writers” and related genes in forest plots using univariate and multivariate Cox regression. **(G)** Kaplan–Meier curves for BC patients in the training cohort and validation cohort from TCGA database. **(H)** DEGs in the volcano plot. Red and blue dots represent upregulated and downregulated genes, respectively. **(I)** Gene set enrichment analysis (GSEA) for the association of hallmarks, biological process, and molecular function with DEGs. **(J)** Analyses for the expression of 20 “writers” in different RMW_Score groups. ns, no significance, *p < 0.05, **p < 0.01, ***p < 0.001. **(K)** Heatmap for the landscape of immune cell types (rows) in patients (columns). Column annotations represent risk group, risk score, Tumor Purity, ESTIMATE Score, Immune Score, and Stromal Score. **(L)** Correlations of RMW_Score with six immune cell types. KEGG, Kyoto Encyclopedia of Genes and Genomes; CNV, copy number variation; BC, breast cancer; TCGA, The Cancer Genome Atlas; DEGs, differentially expressed genes.

Subsequently, more molecular and genetic insights were focused on clustering. Related gene expressions such as CD274 and CTLA4 were upregulated in cluster 1 over cluster 2 except TBX2 (**Figure 2E**). The results from **Figures 2F, G** revealed that PIK3CA accounted for 36%, the highest incidence in cluster 2, whereas TP53 had the highest incidence (51%) of mutations in cluster 1. Of note, compared with cluster 2, tumor mutational burden (TMB) was significantly enriched in cluster 1 (**Figure 2H**). Moreover, 69 differentially expressed pathways in two clusters were identified as related to survival (**Figure 2I**). GSVA was performed to investigate these differentially expressed pathways between the cluster subtypes. In that matter, BC patients with low expression of regulation of autophagy pathway had favorable prognosis, whereas high expressions of JAK/STAT signaling and drug metabolism cytochrome p450 pathways were significantly associated with worse outcomes (**Figures 2J–L**). Other major pathways were exhibited in the heatmap (**Figure 3A**). Furthermore, as displayed in **Figure 3B**, PCIF1 and NSUN2 had a widespread frequency of copy number variation (CNV) gain in both clusters.

### 3.2 Investigation of five types of RNA methylation modification “writers”

The findings based on the cluster subtype supported the roles of RNA methylation modification in prognosis and immune infiltration. To quantify the RNA methylation modification patterns in individual BC patients, we attempted to develop a prognostic score model. The roles of 20 RNA modification “writers” were explored in this work. According to current investigations (19, 23, 30, 31), seven m^6^A modification “writers”, five m^1^A modification “writers”, one m^6^Am modification “writer”, five m^5^C modification “writers”, and two m^7^G modification “writers” were included. First, we observed that compared with normal samples, the expression of “writers” such as TRMT61A, TRPM5, TRMT10C, and NSUN2 was significantly upregulated in BC samples, whereas “writers” like TRMT61B and ZC3H13 were apparently decreased in BC samples (**Figure 3C**). Additionally, pairwise correlations among the expression of the 20 writers were calculated to estimate their relationships (**Figure 3D**). Obviously, a remarkable correlation existed in the same categories. Significant correlations were also observed among different types of RNA modification writers. The expressions of METTL1, TRMT61A, NSUN5, and NSUN3 were positively correlated with other writers. On the contrary, negative correlations were present in the expression of NSUN6, RBM15, NSUN2, TRMT6, TRMT10C, and WTAP with other writers. Thus, negative correlations occurred more frequently than positive ones.

### 3.3 Construction and validation of a prognostic signature for BC

We then performed univariate Cox regression analysis to evaluate the relationship between these writers and the prognosis of BC patients (**Figure 3E**). Further analysis of these writers, 20 writers, and 82 related genes were curated (p< 0.05, Cor≫0.65 . Then, machine learning was utilized to identify three genes (RBM15B, TCP1, and ANKRD36) linked with BC prognosis (**Figure 3F**). In that matter, TCP1 was correlated with m^6^A modification “writer” WTAP. According to the results, a scoring model termed as RMW_Score (RNA methylation modification “writers” and related genes_risk score) was constructed to quantify RNA modification patterns of individual BC patients. The RMW_Score of each patient could be calculated, and 1,089 BC cases from TCGA database were divided into RMW_Score-high and RMW_Score-low groups. Then, the Kaplan–Meier survival analysis was performed, suggesting that patients in the RMW_Score-high group had a worse outcome than the RMW_Score-low group in both the training cohort (n = 545) and validation cohort (n = 544) (p< 0.001, **Figure 3G**).

### 3.4 Immune landscapes of RMW_Score signature

To explore the potential mechanisms underlying the different prognoses of RMW_Score-high and RMW_Score-low groups, we first investigated the DEGs in the two groups. As indicated in the volcano plot (**Figure 3H**), a total of 1,272 DEGs consisting of 1,053 upregulated and 219 downregulated genes were identified. Then, we observed that these genes were enriched in biological processes, such as the interleukin-1-mediated signaling pathway and nucleoside triphosphate metabolic process by GSEA (**Figure 3I**). These DEGs were also found related to hallmarks like mTORC1 signaling and reactive oxygen species pathways. We also compared the expression of 20 “writers” in the two groups (**Figure 3J**) and found that in the RMW_Score-low group, NSUN6, TRMT61B, PCIF1, ZC3H13, METTL14, NSUN4, RBM15B, METTL16, NSUN3, and VIRMA were upregulated while TRMT10C, METTL1, NSUN5, and WTAP were downregulated.

**Figure 4:**
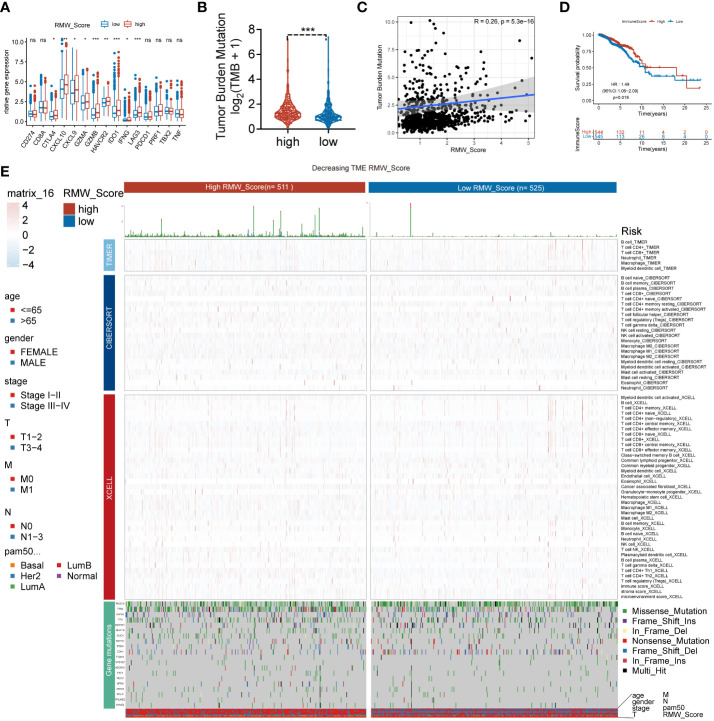
**(A)** The difference in the expression of different immune targets between RMW_Score-high (red) and RMW_Score-low (blue) groups. ns, no significance, *p < 0.05, **p < 0.01, ***p < 0.001. **(B)** The TMB of two RMW_Score groups is compared and plotted. **(C)** The correlation of TMB with RMW_Score is shown. **(D)** Kaplan–Meier curves analyses revealed that high immune scores were associated with more favorable OS (p = 0.019). **(E)** A heatmap designed for the normalized scores of immune and stromal cell infiltrations. Blue, lower infiltration; red, higher infiltration. Wilcoxon test was used to compare the difference between the groups. ^*^p< 0.05, ^**^p< 0.01, ^***^p< 0.001. Meanwhile, gene mutation patterns and clinical characteristics are exhibited as an annotation. TMB, tumor mutational burden; OS, overall survival.

Next, the ssGSEA approach was applied to estimate the infiltrating levels of immune cells in BC. The association of immune cell distribution with different subtypes (RMW_Score, tumor purity, estimate Score, immune score, and stromal score) is depicted in the heatmap (**Figure 3K**). In that matter, the infiltrating levels of major immune cells were positively linked with RMW_Score, including monocyte, gamma delta T cell, and activated CD4 T cell. The correlations of the six types of immune cells (activated CD4 T cell, gamma delta T cell, monocyte, activated CD8 T cell, activated dendritic cell, and CD56bright natural killer cell) with RMW_Score could be viewed in **Figure 3L**. Furthermore, the significant association of RMW_Score with nine immune checkpoints including CTLA4 was observed (**Figure 4A**). We also set sight on the distribution of TMB and found that patients with high RMW_Score had higher TMB (p< 0.001, **Figure 4B**). In addition, a positive correlation was observed between RMW_Score and TMB (**Figure 4C**). The prognosis of BC patients with high immune scores was better than that of patients with low immune scores (p = 0.019, **Figure 4D**). We also investigated the distribution of immune infiltrating cells among RMW_Score-high and RMW_Score-low groups *via* TIMER, CIBERSORT, and xCell (**Figure 4E**). These results suggested the involvement of the RMW_Score signature in the BC immune microenvironment.

### 3.5 Mutation status associated with RMW_Score signature

**Figure 5:**
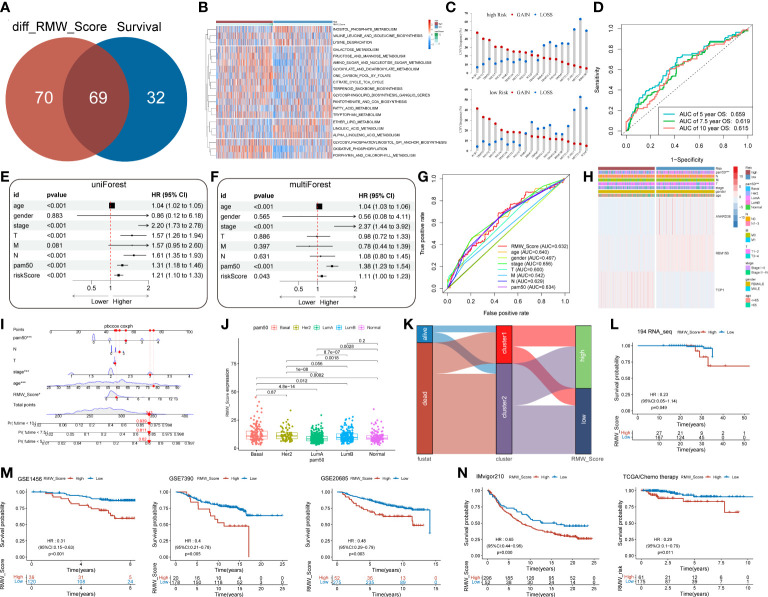
**(A)** Venn diagram for 69 distinct pathways related to survival in two subgroups. **(B)** The survival-related pathways (rows) for each patient (columns) are shown in the heatmap. Red represents high expression, and blue represents low expression. **(C)** The CNV gain and loss frequency in RMW_Score-high and RMW_Score-low groups. **(D)** ROC curve analyses were performed to evaluate the predictive value of RMW_Score in patients (AUC of 0.659, 0.619, and 0.615 for 5-, 7.5-, and 10-year overall survival, respectively). **(E, F)** Forest plots for the independence of RMW_Score as a prognostic predictor by univariate and multivariate Cox regression. **(G)** ROC curves for RMW_Score, age, gender, stage, T, M, N, and PAM50. **(H)** Heatmap depicting the relationships between three patterns (one RNA modification “writer” and two related genes) and RMW_Score and clinical characteristics. **(I)** RMW_Score, age, stage, T, N and PAM50 were used to construct a nomogram (*, p< 0.05; ***, p< 0.001). **(J)** Comparison of RMW_Score expression in different PAM50 subgroups. **(K)** Alluvial diagram of survival status in groups with different clusters and RMW_Score. **(L)** The Kaplan–Meier curves for 194 patients from our follow-up data. **(M)** Kaplan–Meier curves suggested that the RMW_Score-low group all had better OS in the GSE1456 cohort (n = 159, p< 0.001), GSE7390 cohort (n = 198, p = 0.005), and GSE20685 cohort (n = 327, p = 0.003). **(N)** The Kaplan–Meier curves of OS for predicting the immunotherapeutic and chemotherapeutic benefits respectively in the IMvigor210 cohort and TCGA cohort. CNV, copy number variation; ROC, receiver operating characteristic; AUC, area under the curve; OS, overall survival.

Moreover, 69 differentially expressed pathways linked with OS were selected (**Figure 5A**). A heatmap with GSVA was established to visualize and evaluate the major relative pathways, including amino sugar and nucleotide sugar metabolism, oxidative phosphorylation, and galactose metabolism pathways (**Figure 5B**). Of note, CNV mutations existed prevalently. PCIF1, NSUN2, and TRMT6 exhibited an extensive frequency of CNV gain no matter in the RMW_Score-high or RMW_Score-low group, as shown in **Figure 5C**.

### 3.6 RMW_Score signature for nomogram construction

The AUC values of the signature for predicting 5-, 7.5-, and 10-year OS were respectively 0.659, 0.619, and 0.615 (**Figure 5D**). To further confirm the predictive capacity of the RMW_Score signature, we employed univariate Cox regression analysis based on clinicopathological features including age, gender, stage, T, M, N, and PAM50 (**Figure 5E**). It implied that RMW_Score could serve as a prognostic biomarker (HR = 1.210, 95%CI 1.102–1.329, p< 0.001). Moreover, with multivariate Cox regression analysis performed, RMW_Score was an independent and robust predictive factor (**Figure 5F**, HR = 1.109, 95%CI 1.003–1.227, p = 0.043). The AUC of the RMW_Score signature was 0.632, validating its predictive advantage compared with other factors (**Figure 5G**). Additionally, a heatmap in **Figure 5H** showed the association of RNA modification patterns with clinical features and RMW_Score. As expected, a nomogram was constructed according to RMW_Score, PAM50, age, stage, N, and T stage (**Figure 5I**). As **Figure 5J** shows, the distribution of RMW_Score in PAM50 subtypes was different significantly. The alluvial diagram in **Figure 5K** reveals associations between two clusters and two RMW_Score groups in TCGA patients.

### 3.7 External validation and potential therapeutic value for RMW_Score signature

A total of 194 BC samples from our follow-up data were further analyzed (**Figure 5L**). As expected, patients with high RMW_Score had worse outcomes, verifying the reliability of the prognostic model. Consistent with these results, patients in the RMW_Score-low group from the GSE1456 cohort (n = 159, p< 0.001), GSE7390 cohort (n = 198, p = 0.005), and GSE20685 cohort (n = 327, p = 0.003) also showed an obvious survival advantage (**Figure 5M**). We also confirmed the predictive role of RMW_Score in immunotherapeutic benefits for BC patients. Considering the lack of published datasets of BC patients receiving immunotherapy, the urothelial cancer patients (IMvigor210) receiving anti-PD-L1 therapy were used in our study. It was noticed that patients with high RMW_Score in the IMvigor210 cohort had inferior prognoses than those with low RMW_Score (p = 0.030, **Figure 5N**). Moreover, 236 BC patients receiving chemotherapy selected from TCGA dataset were divided into RMW_Score-high and RMW_Score-low groups. The Kaplan–Meier curves showed that patients in the RMW_Score-low group had more survival benefits (p = 0.011, **Figure 5N**). Together, RMW_Score could function as an effective biomarker for managing suitable and satisfactory treatment strategies.

**Figure 6:**
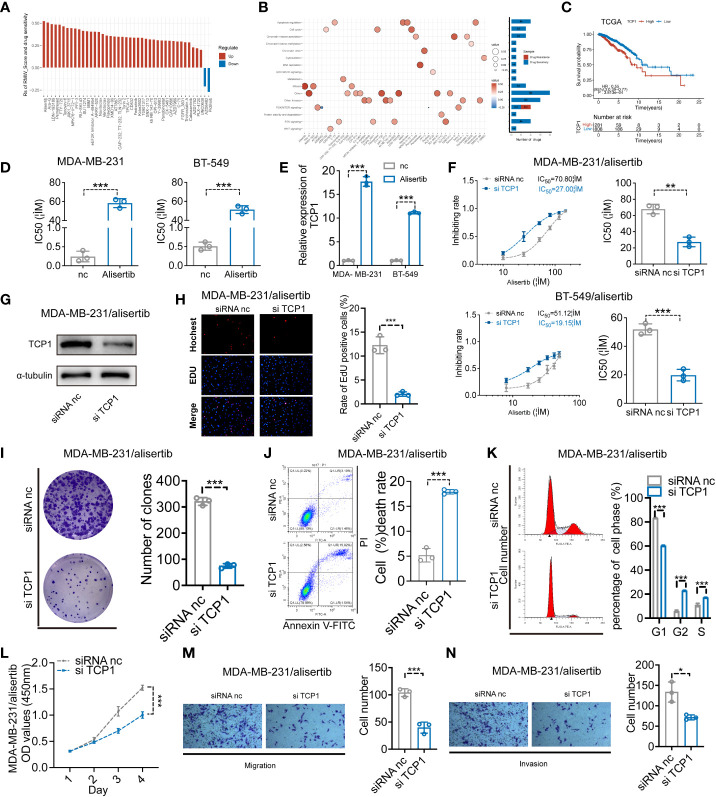
**(A)** Spearman’s analysis estimating the correlation between RMW_Score and drug sensitivity. Each column indicates a drug. Rs > 0, drug resistance; Rs< 0, drug sensitive. **(B)** Drug-targeted signaling pathways based on the RMW_Score. Red represents resistance; blue represents sensitivity. The significance of the correlation was indicated by the size of the point. **(C)** The Kaplan–Meier curves for subgroups with different TCP1 expressions. **(D)** The cell viability assay was used to compare drug resistance between BC cells and their respective drug-resistant cells. **(E)** The expression levels of TCP1 were detected by qRT-PCR. **(F)** The effects of expression of TCP1 on the inhibiting rate were calculated by CCK-8 assay after 48-h treatment of alisertib, and the IC50 was calculated by SPSS. **(G)** The efficiency of TCP1 knockdown was confirmed by Western blotting analysis. **(H)** EdU assays indicated the effects of TCP1 knockdown on cell proliferation in MDA-MB-231/alisertib cells. Scale bars = 100 μm. **(I)** Colony formation assay of siRNA-treated MDA-MB-231/alisertib cells. **(J)** Apoptosis analysis of the effects of TCP1 knockdown and statistical analysis are shown. **(K)** Cell cycle analysis of the effects of TCP1 knockdown and the cell cycle distribution is shown. **(L)** CCK-8 assays showed the effects of TCP1 knockdown on MDA-MB-231/alisertib cell proliferation. **(M, N)** Effects of TCP1 knockdown on cell migration and invasion in MDA-MB-231/alisertib cells by transwell assay. Scale bars = 100 μm. Data were analyzed with the mean ± SD of three replicates. *p< 0.05, **p< 0.01, and ***p< 0.001. BC, breast cancer; CCK-8, Cell Counting Kit-8.

We evaluated the relationship between the RMW_Score and the response to medications in BC cell lines in order to better understand the implications of the RMW_Score on drug response. In the Genomics of Drug Sensitivity in Cancer database, we found 43 substantially linked relationships between RMW_Score and drug sensitivity from 345 drugs by using Spearman’s correlation analysis (**Figure 6A**) (32). In that matter, 41 pairs displayed drug resistance associated with the RMW_Score, including alisertib (Rs = 0.526, p< 0.001), Ara-G (Rs = 0.51, p< 0.001), and LDN-193189 (Rs = 0.492, p< 0.001). Drug sensitivity related to the RMW_Score could be noticed in two pairs, including AZD6482 (Rs = −0.212, p< 0.05) and pictilisib (Rs = −0.273, p< 0.01). As illustrated in **Figure 6B**, drug sensitivity correlated with RMW_Score-high was mainly targeting PI3K/mTOR signaling pathway, whereas drug sensitivity correlated with RMW_Score-low was targeting apoptosis regulation and RTK signaling pathway.

### 3.8 TCP1 expression positively correlated with alisertib resistance

The RMW_Score model of this work was constructed based on RBM15B, TCP1, and ANKRD36, in which TCP1 was the most significant element for its lowest p-value (p< 0.001). The Kaplan–Meier analysis of TCP1 supported that TCP1 was a risk factor for BC patients, and its high expression was apparently associated with poor OS (**Figure 6C**, p< 0.001). Moreover, the potential role of TCP1 in regulating drug resistance of various tumors including acute myeloid leukemia (AML) and lung adenocarcinoma (LUAD) has been investigated in recent studies (33, 34). In our work, the cell viability assay and quantitative reverse transcription polymerase reaction (qRT-PCR) results respectively confirmed that MDA-MB-231/alisertib and BT-549/alisertib cells were more resistant to alisertib and had higher TCP1 expression than their parental cells significantly (**Figures 6D, E**). These findings suggested that TCP1 may contribute to BC cells’ resistance to alisertib. Therefore, we transiently transfected MDA-MB-231/alisertib and BT-549/alisertib cells, which have considerably greater TCP1 expression, with TCP1-specific siRNA vector to reduce TCP1 expression to investigate whether the cellular TCP1 level is connected to the resistance of BC cells to alisertib. According to evidence from cell viability assays, knocking down TCP1 in MDA-MB-231/alisertib and BT-549/alisertib cells increased alisertib’s capacity to suppress cell viability and reduced the drug’s half-life (IC50) (**Figure 6F**). We constructed TCP1 siRNA and verified the knockdown efficiency in MDA-MB-231/alisertib cells through Western blotting (**Figure 6G**).

### 3.9 The effects of TCP1 knockdown in MDA-MB-231/alisertib

For evaluating the effects of TCP1 knockdown in MDA-MB-231/alisertib, a series of experiments *in vitro* were conducted. It was observed that TCP1 knockdown significantly weakened MDA-MB-231/alisertib cell proliferation through EdU (**Figure 6H**). The amount of MDA-MB-231/alisertib colonies was lower in the si-TCP1 group than that in the negative control (NC) group, suggesting that the colony formation capacity could be affected by TCP1 knockdown (**Figure 6I**). Additionally, we discovered that the quantity of apoptotic MDA-MB-231/alisertib cells was dramatically increased in the si-TCP1 group (**Figure 6J**). As shown in **Figure 6K**, TCP1 knockdown increased the percentage of MDA-MB-231/alisertib in the S and G2 phases while decreasing the G1 phase population by flow cytometry analyses. Furthermore, the result in **Figure 6L** indicated the effect of TCP1 on promoting the MDA-MB-231/alisertib cell proliferation through CCK-8 assays. We also found that compared with the NC group, the si-TCP1 group was less invasive and migrated (**Figures 6M, N**). Collectively, these data revealed the effects of TCP1 on promoting the proliferation and migration of MDA-MB-231/alisertib and drug resistance.

## 4 Discussion

Increasing evidence has revealed the essential roles of RNA methylation modifications in the TME through interaction with numerous “writers”. The single type of RNA methylation modification “writer” was the main concern in most reported works of literature. However, a clear view of the functions of multiple types of “writers” in BC has not been established. Herein, the RMW_Score model (containing m^6^A, m^1^A, m^6^Am, m^5^C, and m^7^G) was developed *via* machine learning approaches as an integrative predictor of BC patients’ survival and therapeutic responses in BC. Then, using data from 20 RNA modification enzymes, we determined two unique RNA modification patterns and identified two BC subtypes associated with RNA modification. The further landscape of immune cell, genetic, and molecular characteristics in the two clusters was explored, and cluster 2 was closely correlated with higher infiltrating levels of immune cells compared with cluster 1. We also found that 69 pathways differently expressing in the two clusters were related to survival, most of which like the JAK–STAT signaling pathway appeared to upregulate in BC patients with favorable clinical outcomes. These findings revealed the essential roles of RNA methylation modifications in the TME and BC prognosis.

Tumor cell metastasis and drug resistance are impacted by epithelial–mesenchymal transition (EMT) (35, 36), and naïve CD4^+^ T-cell recruitment might inhibit TI Tregs, restore immune tumor killing, and inhibit cancer growth and metastases (37). According to Bach et al., the JAK–STAT pathway, which regulates the expression of PDL1 and MHC class I, is activated by IFN-γ released from effector T cells in cancer cells. This signaling cascade can cause tumor cell death in a variety of ways (38, 39). Therefore, we developed the RMW_Score scoring model (consisting of one distinct RNA methylation modification pattern (RBM15B) and two related genes (TCP1 and ANKRD36)) to quantify RNA methylation modification patterns in individual tumors, predicting the prognosis of BC patients and facilitating effective therapeutic strategies. RBM15B, the paralog of RBM15, was found involved in prognostic models of several types of tumors, such as alcohol-related hepatocellular carcinoma (A-HCC), small cell lung cancer (SCLC), and melanoma (40–42). The predictive values of TCP1 and ANKRD36 were also elucidated in many investigations (43, 44). For example, it was reported that TCP1 may contribute to HCC cell proliferation and metastasis by regulating the Wnt7b/β-catenin pathway and is a molecular marker for the prognosis (44). Of note, we explored the involvement of the RMW_Score model. The RMW_Score-high subtype indicated poor survival significantly, which was verified in several cohorts including the GSE1456 cohort, GSE7390 cohort, GSE20685 cohort, and 194 samples from follow-up data.

Subsequently, we observed the association of the RMW_Score model with the tumor microenvironment and found that the amounts of immune infiltrating cells were apparently different between the two groups. The RMW_Score-low group was associated with high infiltrating levels of memory B cell, eosinophil, mast cell, natural killer cell, and plasmacytoid dendritic cell. Recent studies reported that tumor-infiltrating B cells (TIL-B) in BC were correlated with improved outcomes (45, 46). An analysis of HER2+ and triple-negative breast cancer patients from the BIG 02-98 clinical trial confirmed the correlation between positive outcomes and higher TIL-B densities (46). Similar to these studies, the results in our work implied that high amounts of memory B cells were associated with better clinical outcomes.

Additionally, the results from enrichment analyses revealed that DEGs were remarkably enriched in the interleukin-1-mediated signaling pathway, nucleoside triphosphate metabolic process, mTORC1 signaling, and reactive oxygen species pathway. In a further analysis of these pathways, we found 69 pathways related to survival, such as Notch signaling and oxidative phosphorylation (OXPHOS) pathways. Recent studies indicated that OXPHOS could be upregulated in many cancers, including lymphomas, pancreatic ductal adenocarcinoma, and breast cancer, based on which OXPHOS inhibitors exerted positive effects on treating cancers (47). For instance, marizomib (Mzb) inhibited TNBC metastasis *via* OXPHOS inhibition (48). Moreover, we found that BC cases from either the RMW_Score-low group or the RMW_Score-high group had high mutations of TP53 and PIK3CA and an enormous frequency of CNV gain of PCIF1, NSUN2, and TRMT6. Nevertheless, patients with high RMW_Score were dramatically associated with TMB.

Moreover, the RMW_Score model could independently predict the prognosis of BC patients revealed by univariate and multivariate Cox regression analyses, thus showing predominant advantages compared with several clinicopathological parameters. Based on RMW_Score, PAM50, age, stage, T, and N stage, a visualized nomogram was designed to offer risk score prediction for each patient. Furthermore, we also found the remarkable effects of the RMW_Score model on therapeutic response. RMW_Score was correlated with sensitivity to drugs (AZD6482 and Pictilisib) targeting PI3K/mTOR signaling pathway and resistance to drugs targeting apoptosis regulation and RTK signaling pathway. It was discovered that the drug metabolism enzyme P450 plays a significant role in affecting the development of BC. The results demonstrated that patients in the RMW_Score-high group may benefit from drugs targeting PI3K/mTOR signaling pathway. In addition to drug response, we analyzed the effects of RMW_Score as a predictor of chemotherapy and immunotherapy responses. As expected, patients achieving chemotherapy with low RMW_Score presented prolonged survival outcomes. Urothelial cancer patients (IMvigor210) in the RMW_Score-low group achieved apparent benefits from anti-PD-L1 therapy. Despite the veracity of RMW_Score in anti-PD-L1 therapy, further work is needed to assess the response in patients with BC. Together, our model could work as a robust and precise biomarker for improving therapeutic strategies.

T-complex protein 1 (TCP1), also termed CCT1, is one of eight CCT (chaperonin-containing T complex) subunits (CCT1–8). According to recent investigations, TCP1 could promote the tumorigenesis and progression of various tumors (49, 50). For instance, TCP1 has been found overexpressed in ovarian cancer, which is correlated with poor prognosis (49). Activation of the PI3K signaling pathway may contribute to the process of TCP1 upregulation in promoting cancer progression. TCP1 is also found crucial to BC patients’ survival, connected to driving oncogenes (50). Furthermore, increasing studies have reported the effects of TCP1 involved in drug resistance (34). TCP1 presented high expression in acute myeloid leukemia cells, responsible for drug resistance. In the current work, we found that TCP1 was elevated in drug-resistant MDA-MB-231/alisertib and BT-549/alisertib cells. While TCP1 overexpression imparts resistance to the therapeutic benefits of MDA-MB-231/alisertib and BT-549/alisertib, TCP1 downregulation can increase their sensitivity to alisertib. In light of these findings, it appears that alisertib drug resistance was linked to high TCP1 levels.

Although the RMW_Score model presented reliable and efficient prediction in prognosis and therapeutic response of BC patients, there still existed some limitations. Tumor heterogeneity and retrospective datasets were the main reasons. Moreover, anti-PD-L1 therapy response was analyzed in this work based on patients with urothelial cancer (IMvigor210) due to insufficient data on BC patients undergoing anti-PD-L1 therapy. Thereby, further investigation is needed to evaluate immunotherapeutic response in BC patients.

## 5 Conclusions

In summary, RBM15B, TCP1, and ANKRD36 were identified by machine learning approach to construct RNA methylation modification “writers” and related genes_risk score (RMW_Score) model. We found that RMW_Score could predict BC patient survival and therapeutic benefits as a sensitive marker. Additionally, TCP1 was confirmed to promote BC alisertib-resistant cell proliferation and migration *in vitro*.

## Data availability statement

The datasets presented in this study can be found in online repositories. The names of the repository/repositories and accession number(s) can be found in the article/supplementary material.

## Author contributions

XZ, JT and LS conceived and designed the study. XZ, YZ and LS performed bioinformatics analyses. YZ, CZ and HZ analyzed and interpreted data and/or supervised parts of the study. XZ and LS wrote the paper. JT and YZ revised the paper. All authors contributed to the article and approved the submitted version.
